# Effectiveness of three treatment strategies on occupational limitations and quality of life for patients with non-specific chronic low back pain: Is a multidisciplinary approach the key feature to success: study protocol for a randomized controlled trial

**DOI:** 10.1186/1471-2474-15-131

**Published:** 2014-04-16

**Authors:** Audrey Petit, Ghislaine Roche-Leboucher, Luc Bontoux, Valérie Dubus, Yohann Ronzi, Yves Roquelaure, Isabelle Richard

**Affiliations:** 1 LUNAM University, Laboratory of Ergonomics and Epidemiology in Occupational health, (LEEST), University of Angers, Faculty of Medicine, Rue Haute de Reculé, Angers cedex 01 49045, France; 2 Occupational Health Department, CHU of Angers, 4 Rue Larrey, Angers cedex 09 49933, France; 3 Department of Physical Medicine and Rehabilitation, CHU of Angers Rue des Capucins, Angers 49000, France

**Keywords:** Chronic non-specific low back pain, Multidisciplinary rehabilitation, Private physiotherapy, Mixed strategies

## Abstract

**Background:**

Chronic low back pain (cLBP) is a significant public health problem, being the primary cause of work absenteeism, as well as affecting sufferers’ quality of life, in industrialized society. International guidelines recommend intensive multidisciplinary approaches for patients with cLBP. However, these costly and time-consuming programs can only be offered to a minority of the most heavily affected patients and therefore do not seem likely to respond to public health requirements. Lighter programs may be an alternative to full time hospital-based programs with valuable results in terms of disability and occupational activity for cLBP patients. It is therefore important to define both what the determining components of management to improve activity restriction are and how to treat a larger number of patients more effectively at a lower cost. The aim of this study is to compare three programs with various levels of intensity and multidisciplinary.

**Methods/Design:**

This paper describes the protocol for a prospective, randomized, controlled, clinical trial in working aged patients with cLBP. Three treatment strategies are compared: (1) intensive and multidisciplinary program conducted in a rehabilitation center; (2) less intensive outpatient program conducted by a private physiotherapist; (3) mixed strategy combining the same out program with a multidisciplinary intervention. The primary outcome of the trial is the impact of the mixed strategy on being able to work compared to hospital centered-program and out program. The secondary outcome is the impact of the mixed strategy on quality of life and social ability compared to the two others programs. The intervention part of the trial programs will take 5 weeks and observational follow-up will take 12 months. The sample size will be 180 participants (60 for each arm). The project has been approved by the Ethical Committee of Angers Hospital, France.

**Discussion:**

On the hypothesis that a multidisciplinary approach is the key feature to programs success in reducing social and occupational impairment in cLBP patients, we suggest that it is possible to achieve the same results with less intensive strategies if a multidisciplinary approach is maintained.

**Trial registration:**

Current Controlled Trials NCT02030171.

## Background

Chronic low back pain (cLBP) is a significant public health problem, being an important cause of work absenteeism, as well as affecting sufferers’ quality of life [1,2]. LBP remains the primary cause of absenteeism and disability in every industrialized society and patients who develop cLBP (pain and disability persisting for more than 12 consecutive weeks) use more than 80% of all health resources for back pain [3].

International Guidelines recommend the use of supervised active exercises, multidisciplinary approaches, cognitive-behavioral therapies and measures of social and professional order for patients with cLBP [1,4,5]. Referral for these programs is preferred for patients with cLBP on prolonged sick leave [1,5,6] and treatment management is increasingly focused on the prevention of activity improvement and ability restrictions, and have as an explicit goal return to or staying at work, regardless of changes in the painful condition. The impact of functional recovery has been assessed in terms of pain, disability and occupation [7-17]. Although pain outcomes differ between studies, action to improve functional and occupational capacity are consistently encouraging, particularly in France [11,14,16-18]. However, these costly and time-consuming intensive multidisciplinary programs can only be offered to a minority of the most heavily affected patients and therefore do not seem likely to respond to public health requirements.

Lighter programs may be an alternative to full time hospital-based programs at the same stage of treatment strategy with valuable results in terms of disability and occupational activity for cLBP patients [16,17,19-22]. It is therefore important to define both what the determining components of management to improve activity restriction are, including occupational status, and how to include more hospital-independent programs in our health care system to treat a larger number of patients more effectively at a lower cost.

This study follows two previous studies by our team:

- The first [16,23] prospectively evaluated an intensive multidisciplinary Functional Restoration Program (FRP). A cohort of 87 patients had been treated and followed up two years after treatment and the results revealed a significant reduction (60%) in the number of days’ sick leave during the two years following inclusion.

- The second [22] randomly compared the same FRP to less intensive Ambulatory-based Individual Physiotherapy (AIP): 64 patients were included in the FRP arm and 68 patients in the AIP arm. This study demonstrated the superiority of the first program in terms of number of days’ sick leave during the 6 months following treatment. The difference was also highly significant with regard to outcomes assessing physical capacity, but there was no between-arm difference for pain intensity or quality of life criteria. The hypothesis proposed to explain this result was that the crucial difference between the two programs was the multidisciplinary nature of the approach, and we considered that it would be possible to achieve the same results with less intensive AIP if this multidisciplinary approach was maintained. Obtaining such information would potentially allow a significant increase in the number of patients offered a multidisciplinary strategy by increasing treatment availability and decreasing program costs.

### Aim of the study

On the hypothesis that a multidisciplinary approach is the key feature to FRP success in reducing social and occupational impairment in cLBP patients, we suggest that it is possible to achieve the same results with less intensive AIP strategies than FRP if a multidisciplinary approach is maintained. The aim of this study was to compare three treatment strategies to demonstrate this hypothesis:

- Intensive and multidisciplinary FRP conducted in a rehabilitation center;

- Less intensive outpatient AIP conducted by a private physiotherapist member of a care network;

- Mixed strategy combining the same AIP with multidisciplinary intervention.

### Hypothesis regarding the primary aims

The expected benefits for subjects participating in the study are improvement in their quality of life and improved remaining at work, resulting in a decrease in the number of days’ sick leave, and reducing the risk of progressive exclusion from work.

The main results expected from the study are (1) a significant difference between the mixed strategy and AIP in the number of days’ sick leave and quality of life and (2) no significant difference in these criteria between FRP and the mixed strategy despite any difference in outcomes evaluating only impairments and activity limitations. Such results would show that a multidisciplinary approach is the key feature to the success of such programs.

## Methods/Design

### Study design

**Figure 1:**
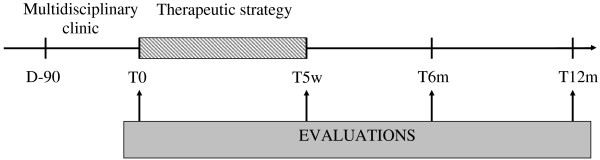
Clinical study design.

This is a mono-center study with a prospective randomized controlled design, comparing the effectiveness of three treatment strategies. Participants with non-specific cLBP are being randomized into one of the three arms of the study, with a follow-up of 1 year (see Figure 1). The randomized controlled trial (RCT) includes both effectiveness and an economic study.

### Setting

Participants are referred to a French cLBP care network to support medical and occupational issues. This network includes a multidisciplinary cLBP clinic with a rehabilitation physician, an occupational health practitioner (OP), a psychologist and an occupational health nurse. Rehabilitation programs are provided in a rehabilitation center or by one of the private physiotherapists of the network. After attending the multidisciplinary LBP clinic and randomization, participants are subsequently referred to the rehabilitation center or to a private physiotherapist of the region or both, depending on the arm of the trial. The study is expected to last three years and will stop once completed, ie at the end of the last participant’s follow-up.

### Ethical aspects

This investigation is being conducted according to the international recommendations for cLBP management [1,4,5]. All patients are asked to provide informed written consent prior to randomization, using standard forms. Information for the participants and their agreement to study participation is considered “informed consent”. It is pointed out to the participants that study participation is voluntary and that they may refuse to participate or discontinue participation at any time without disadvantages or loss of benefits. According to the national data protection laws, all personal data are treated as confidential and used only for scientific purposes. Ethical approval has been granted by the French Ethics Committee.

### Participants

Subjects of working age suffering from non-specific cLBP diagnosed according to generally accepted scientific criteria are acceptable for enrolment [1,24].

#### Inclusion criteria

Patients may be included if they are able to sign the informed consent form, and are aged between 18 and 55 years, with non-specific LBP lasting for at least 3 months without improvement, LBP leading to at least 1 month’s sick leave during the preceding year and/or 3 months’ sick leave during the preceding two years, and an open-ended or fixed-term contract of work in the public or private sector.

#### Exclusion criteria

Patient who are unable to give informed consent, have a disability preventing them attending physical therapy with a physiotherapist of the study, with diagnosed malignant, traumatic, infectious or inflammatory LBP, acute sciatica, symptomatic lumbar spinal canal stenosis or progressive spondylolisthesis, cardiac or respiratory insufficiency (diagnosed after exercise stress test on bicycle ergometer), articular or neurologic impairment incompatible with a physical exercise program (but not uncomplicated depression), psychiatric disorders precluding participation in group therapy, pregnancy or breast-feeding, legal protection for vulnerable individuals, lack of health insurance are excluded.

#### Withdrawal criteria

A patient may leave the study at his own or an investigator’s initiative. The exclusion criteria are expected to extent to withdrawal of consent for participation, development of a criterion for non-inclusion or administration of a prohibited treatment during the 5 weeks’ course.

### Screening for participation and randomization

Participants are selected by the study investigators from patients referred to the regional cLBP network. When a patient is referred to the regional cLBP network, the rehabilitation physician screens information provided by the referring general practitioner or medical specialist before referring him to the multidisciplinary cLBP clinic. Each patient undergoes a standardized multidisciplinary evaluation including a medical examination, psychological, social and occupational interviews and self-administered questionnaires. The rehabilitation physician verifies whether an extensive physical examination and imaging or laboratory studies according the guidelines [24] have been performed if necessary. The rehabilitation physician explains the study procedure and, if someone meets the inclusion criteria and has none of the exclusion criteria, asks the patient to sign the informed consent form. After signing, one of the three treatment strategies is randomly allocated to each patient.

The randomization procedure of participants is determined by a computer randomization feature which automatically allocates the patient to one of the treatment strategies. This system has been described in previous studies [16,17,22]. An independent research assistant prepares envelopes and numbers them sequentially according to the randomization list. Envelopes are given to the rehabilitation physician who opens them at the end of the clinical evaluations described above.

### Interventions

The three treatment strategies last 5 weeks but the treatment content of each strategy is different. FRP involves 6 hours of treatment a day, 5 days a week in group of 6 to 8 patients. AIP includes individual rehabilitation with a private physiotherapist for 1 hour 3 times a week and individual exercises to be performed at home twice a week for 50 minutes. The mixed strategy includes AIP combined with 5 one-day group sessions. Patients are off work during the 5 weeks of treatment in all three groups.

#### Functional restoration program (FRP)

The group performs exercises supervised by a physiotherapist who adjusts the exercise intensity to each participant every week. During the first week, patients learn muscular warm-up and stretching techniques, improve their flexibility, and perform cardio-respiratory exercises. During the second week, patients begin muscular-strengthening exercises. During the third week, muscular strengthening increases with endurance exercises. Patients perform weightlifting as well as proprioception and coordination exercises. In the fourth and fifth weeks, the intensity of strengthening exercises increases progressively. The endurance training is adapted to each patient’s heart rate and to the exercise stress test performed before the program. Patients perform work simulations during occupational therapy sessions.

Strengthening exercises are performed exclusively with isotonic techniques. Proprioception is developed with static and dynamic destabilization exercises. Walking, running, and cycling develop cardio-respiratory endurance.

Each week, patients attend a clinic with the rehabilitation specialist who supervises the program. They are referred to the psychologist at least once in the first week and for further treatment if requested. Dietary advice is given. The schedule of interventions is standardized for all patients (Additional file 1).

#### Ambulatory individual physiotherapy (AIP)

Each individual session lasts 1 hour and includes only active exercises supervised directly by a private physiotherapist of the regional cLBP network. All private physiotherapists participate in an information session and agree to apply the program as described. During the first 2 weeks, the program includes flexibility training and pain management, stretching, and proprioception exercises. Patients continue these exercises during the third and fourth weeks and start muscular strengthening. The last week focuses on functional exercises and endurance training. The program includes 50 minutes of individual home exercises 2 days a week (these can include stretching, jogging and swimming). This part of the program is agreed upon by the patient and physiotherapist and depends on the facilities available. It is not standardized. All exercises are isotonic, and no specific equipment is required or provided. The cardio-respiratory exercise training should be based on 70% of the theoretical maximum heart rate: patients should therefore monitor their heart rate during exercises. At the beginning of the program, patients sign an agreement to follow the prescribed exercises. Patients have to record the duration, type, and number of exercises performed at home.

#### Mixed strategy

The mixed strategy includes two aspects:

1) A weekly review one day a week in a rehabilitation center which combines:

- a 1 hour group session led by a physical rehabilitation specialist. It provides assessment of cLBP perception and discussion of representations and beliefs. It is a place of exchange between the patient group and creation of a “group effect”;

- a group meeting with the dietician (specifically trained in therapeutic education) and the sports therapist. This meeting includes monitoring and discussion of physical activities independent for each patient. The sports therapist can advise on appropriate activities. No physical activity is carried out during these one-day sessions.

- psychological support: this consists of a group relaxation session led by a physiotherapist trained in this technique. An individual meeting with a psychologist is systematically offered.

- establishment of a logbook in which the patient records the type and duration of individual physical activity, a summary review of the week, and the difficulties encountered.

2) An ergonomic intervention in the workplace and systematic contact with the OP. Ergonomists intervene in a company after agreement has been obtained with the employer. This procedure involves a standardized evaluation of work postures, a meeting between the supervisor and/or employer, the patient and the occupation health practitioner. A proposal for further intervention for the improvement of working conditions is made when it is appropriate and possible. A consultation is systematically organized with the OP early in the program if this has not already occurred during the preceding three months.

3) Specific coordination with the general practitioner (GP): the rehabilitation specialist contacts each patient’s GP at the beginning and end of the program, and proposes a twice yearly phone appointment to discuss the patient’s progress. Patients’ follow-up appointments with their GPs are scheduled quarterly.

- individual ambulatory physiotherapy, supervised by a private physiotherapist of the cLBP regional network for 3 sessions per week for 5 weeks, using the same protocol and the same conditions as for the AIP strategy;

- multidisciplinary approach, coordinated by the cLBP regional network linked with all partners. This multidisciplinary approach includes:

#### Concomitant treatments

All other treatments are allowed, except for the following: physiotherapy other than AIP in the cLBP regional network, other manual techniques, surgery for back pain, and hospitalization for LBP. In the case of administration of any of these treatments, patients are eliminated from the study.

### Outcome measurement

Standardized questionnaires are used to measure the primary and secondary outcomes. Our choice of outcome evaluation methods is based on two previous trials conducted by our working group to evaluate the effectiveness of various interventions in the management of cLBP [16,17,22]. They are also in line with international recommendations.

#### Primary outcome measurement

The primary outcome of the trial is represented by the effectiveness of the impact of the mixed strategy on being able to work compared to FRP and AIP. Being fit for work is evaluated by the duration of sick leave in 1 year: the number of days’ sick leave during the preceding 12 months and during the 12 months of the study is collected for each patient and verified by checking with the health insurance provider.

#### Secondary outcome measurement

The secondary outcome of the trial is represented by the impact of the mixed strategy on quality of life and social ability compared to FRP and AIP. Quality of life and social ability are evaluated by the Short Form Health Survey questionnaire (SF-36) and the Dallas Pain Questionnaire (DPQ).

- The SF-36 is a self-administered questionnaire used to assess quality of life related to health [25,26]. This tool consists of 36 items grouped into eight dimensions, each corresponding to a different aspect of health: Physical Functioning; Role Limitations due to physical problems; Bodily Pain; General Health perceptions; Energy/Vitality; Social Functioning; Role Limitations due to emotional problems; and Mental Health. These 36 items allow evaluation with two summary scores: Physical and Mental Health Component Summary Scores (assessed by a score between 0 and 100, with higher scores indicating better quality of life).

- the Dallas Pain Questionnaire (DPQ) [27,28] is a self-administered questionnaire used to assess the amount of cLBP that affects four aspects (daily and work-leisure activities, anxiety-depression, and social interest) of patients’ lives. These four aspects are scored from 0 to 100% (higher scores indicating poorer quality of life).

#### Complementary outcome measurement

Criteria evaluating impairments and activity limitations, personal beliefs and environmental conditions are collected because they can be explanatory factors of the primary and secondary criteria [29-39]. They are not endpoints of treatment strategies. All these complementary outcome measurements are shown in Table 1.

**Table 1:** Complementary outcome measures

**Outcomes**	**Criteria**	**Tools**
Impairments and activity limitations	Pain	Visual analog scale [32,34]
	Trunk flexibility	Finger-floor distance [31]
	Trunk muscles endurance	Time of isometric contraction of the flexor muscles of the spine: Ito-test [33]
		Time of isometric contraction of the extensors muscles of the spine: Sorensen-test [30]
	Loading capacity	PILE-test (Progressive Isoinertial Lifting Evaluation) [35,36]
	Anxiety and depression	Hospital Anxiety and Depression scale [38].
Personal beliefs and environmental conditions	Patient’s fears and beliefs for physical activity by	Fear Avoidance Beliefs Questionnaire [37]
	Evaluation of the job constrain	Work Ability Index questionnaire (WAI) [39]

#### Cost analysis

Treatment costs and additional expenses are assessed from the point of view of the health insurance organisation which is paying. This is done in 3 ways:

- a “patient diary” is given to the patient at each follow-up appointment. The patient is asked to note all sickness absences in this diary whether related to cLBP or not, all hospitalizations, medical consultations, investigations, medical treatments and paramedic treatment. This diary is collected by the investigator at each follow-up appointment (end of treatment, 6 months and 1 year).

- the general practitioner is asked to complete and check the filling in the “patient diary”.

- the quality of the health economic data is randomly monitored for 20% of study participants. A request has been made to the each health insurance organization to have repayments continue during each patient’s participation in the study.

#### Compliance with treatment strategies

To verify patient compliance with the treatment management, private physiotherapists participating in the study, general practitioners and institutional therapists report any major difference from the study protocol to the investigators of the study.

### Assessment and procedures

The accuracy and completeness of the data collected is checked weekly by an independent research assistant to provide a suitable level of quality for the study. In particular, controls review the quality and completeness of outcomes, treatment execution and monitoring modalities, the dropout rate, and correct respect of application of enrolment criteria.

#### Inclusion

Inclusion of patients is decided during the multidisciplinary cLBP clinic described above. All patients satisfying inclusion and non-inclusion criteria are offered the possibility to take part in the study. The inclusion appointment includes: verification of inclusion and non-inclusion criteria, information to the patient about the study and signing of informed consent for participation in the study by the patient. Once consent for participation has been signed, the participant is then randomized and allocated to one of the three treatment arms. The treatment strategy is then implemented within the next 90 days and the patient’s schedule is decided (date of initial assessment, appointment dates corresponding to the allowed treatment strategy). Participants are off work for the 5 weeks of treatment.

#### Observational follow-up

Assessments are undertaken in the rehabilitation center by an independent physiotherapist. Four stages of participant assessment are planned in this RCT (Figure 1):

- initial (T0) and end of treatment (T5w) assessments are performed during the seven days preceding the treatment and on the last day of treatment, respectively. These assessments include personal data, medical history, intensity of pain on the VAS, FFD, Ito, Sorensen and PILE tests, DPQ, FABQ, SF-36, HAD and WAI questionnaires.

- follow-up assessments are performed at 6 months and 12 months during the follow-up period. These assessments include interviews and clinical examination, intensity of pain on the VAS, FFD, Ito, Sorensen and PILE tests, DPQ, FABQ, SF-36, HAD and WAI questionnaires, number of days’ sick leave, patient diary (medical and paramedical interventions).

### Adverse events

The study provides a direct benefit for each patient in that all patients receive standardized physiotherapy treatment which is consistent with international recommendations and has already been assessed in our previous studies. No specific adverse effects related to the study are expected for patients. Common adverse events associated with this kind of treatments are those injuries which might occur following the proposed activities, at the rehabilitation center, in the physiotherapist office, or related to transport. Any adverse events would be managed in accordance with current rules of good clinical practice. All adverse events will be reported to the regulatory authority and the Ethics Committee in accordance with relevant regulations.

### Analyses

#### Sample size

Based on the available literature [7-15] and following previous trials [16,17,22], we assume that a difference of 30 days’ sick leave a year between treatment arms is clinically significant. With a sample size of 60 patients in each treatment arm, accepting an alpha error of 0.05 and a power of 0.80, it is possible to measure a minimum difference of 30 days’ sick leave during the subsequent 12 months’ treatment. To compensate for an estimated 10% dropout rate, a total of 180 people will be included.

#### Analyses of efficacy

The effects of treatment conditions on the various outcomes will be compared using an “intention to treat” approach. Data analysis will be performed using SPSS 12.0 software. The significance level (p) has been defined as 0.05. Results will be expressed as mean ± standard deviation (for quantitative data) or percentage (for qualitative data). The initial comparability of the three groups will be verified by ANOVA or Kruskal-Wallis test (if necessary) for quantitative data, and t test for qualitative data. Between groups quantitative data will be compared by Student’s test and qualitative data by Pearson χ2 test or Ficher’s exact test for small samples. Changes within groups will be assessed by t test and MacNemar χ2 for paired data for quantitative and qualitative data, respectively. There will be a post-hoc analysis of the dropout group and missing values. Patient characteristics of the dropouts will be compared to those of the group that complete each treatment.

#### Economic analyses

The health-care costs of each treatment will be evaluated and the cost of each of the three strategies will be compared from the point of view of health insurance, i.e. the payer: sick leave costs will be estimated according to reimbursement of health insurance; costs of hospitalizations and drugs will be assessed using their basic tariffs; costs of medical and paramedical consultations and additional tests will be assessed using the basic tariffs for hospitals and fees for independent practitioners. Costs of each treatment will be evaluated from the point of view of health insurance.

## Discussion

The purpose of this RCT is to compare the effects and the economic aspects of three treatment strategies for patients with non-specific cLBP. The aim of the study is to evaluate the impact on quality of life and ability to work of an original mixed treatment program combining ambulatory-based rehabilitation and hospital-based multidisciplinary intervention.

The findings of this study might help to confirm our hypothesis that the multidisciplinary aspect and not the intensity is the key feature of cLBP management and might therefore allow the development of modified ambulatory-based rehabilitation programs for cLBP. The findings might assist treatment providers, therapists, and people with cLBP to make rational decisions about treatment by enabling us to choose between programs with varying degrees of intensity of physical fitness but maintaining a multidisciplinary intervention.

The consequences of these findings for the application of validated strategies in large populations of people with cLBP are important and would also open up prospects of research for other diseases for which coordination and a multidisciplinary approach can be a more determining factor rather than the intensity of each isolated treatment.

This study has certain limitations. The participants and practitioners cannot be blind. The extent of the home-exercise program is totally dependent on the motivation of the participant to perform the given exercise program and this could influence the outcomes of the study. We aim to increase motivation and involvement in the program by asking the patient to sign a written agreement and to report the actual duration and type of exercises performed.

### Trial status

At the time of the submission of the manuscript, data collection is ongoing. Data will be analyzed between May and October 2014. The results of the trial will be available in November 2014 and published once the analyses are complete.

## Abbreviations

AIP: Ambulatory-based individual physiotherapy; cLBP: Chronic low back pain; DPQ: Dallas pain questionnaire; FABQ: Fear-avoidance beliefs questionnaire; FFD: Fingertip-to-floor method; FRP: Functional restoration program; HAD: Hospital anxiety depression; LBP: Low back pain; OP: Occupation health practitioner; PILE: Progressive isoinertial lifting evaluation; RCT: Randomized controlled trial; SF-36: Short fort health survey questionnaire; VAS: Visual analog scale; WAI: Work ability index.

## Competing interests

All the co-authors declare they have no competing interests.

## Authors’ contributions

AP drafted the manuscript and is the further responsible for the study organisationand oversees patient’s enrollment and data management. GRL, LB, VD, IR and YRoq contributed substantially to literature search, the development of the research questions and the choice of assessment tools, the study design and the study protocol in general. YRon has been involved in drafting the manuscript. YRoq critically revised the manuscript for important intellectual content. Every author read and approved the final version of this paper.

## Pre-publication history

The pre-publication history for this paper can be accessed here:

http://www.biomedcentral.com/1471-2474/15/131/prepub

## Supplementary Material

Additional file 1 Schedule of interventions in the Functional Restoration Program (FRP). Click here for file
