# Supplementary material for: Effectiveness of three treatment strategies on occupational limitations and quality of life for patients with non-specific chronic low back pain: Is a multidisciplinary approach the key feature to success: study protocol for a randomized controlled trial
Source: BMC Musculoskelet Disord. 2014 Apr 16;15:131. doi: 10.1186/1471-2474-15-131 (PMC4031373; doi:10.1186/1471-2474-15-131)
Supplement: Additional file 1 — Schedule of interventions in the Functional Restoration Program (FRP). [file 1471-2474-15-131-S1.docx]

**Additional file 1: schedule of interventions in the Functional Restoration Program (FRP)**

Group interventions (6 to 8 patients):

9:00 AM to 10:00 AM: Warm-up, stretching, and proprioception exercises: walking, running, stretching of trunk and limbs muscles, balance exercises (for dynamic destabilization exercises), and various games.

10:15 AM to 11:15 AM: Strengthening exercises (isotonic training of all major muscular groups).

11:30 AM to 12:30 PM: Aerobic activities (jogging, ball games).

Lunch.

1:30 PM to 2:00 PM: Warm-up, stretching, and proprioception exercises.

2:00 PM to 3:15 PM: Occupational therapy (training in flexibility, endurance and coordination exercises, weight lifting, and work simulation).

3:30 PM to 4:15 PM: Global strengthening exercises and endurance training (jogging, stepping, and cycling exercises).

4:30 PM to 5:00 PM: Balneotherapy (for muscular recovery and proprioception exercises).

Individual interventions: meetings with the physiatrist, the psychologist, and the dietician.
